# A Portable Colorimetric Biosensor Platform for Urinary Colorectal Cancer Biomarker Testing in Low-Resource Settings

**DOI:** 10.3390/bios16090485

**Published:** 2026-09-02

**Authors:** Prashanthi Kovur, Scott MacKay, Songtian Bai, James Cook, Claudia Torres-Calzada, Dipanjan Bhattacharyya, Upasana Singh, David S. Wishart

**Affiliations:** 1 Department of Biological Sciences, CW-405, Biological Sciences Building, University of Alberta, Edmonton, AB T6G 2E9, Canada; kovur@ualberta.ca (P.K.); samackay@ualberta.ca (S.M.);; 2 Department of Electrical and Computer Engineering, University of Alberta, Edmonton, AB T6G 2H5, Canada; 3 Department of Computing Science, University of Alberta, Edmonton, AB T6G 2E8, Canada; 4 Department of Laboratory Medicine and Pathology, University of Alberta, Edmonton, AB T6G 2B7, Canada; 5 Faculty of Pharmacy and Pharmaceutical Sciences, University of Alberta, Edmonton, AB T6G 2H7, Canada; 6 Women and Children’s Health Research Institute, University of Alberta, Edmonton, AB T6G 1C9, Canada

**Keywords:** colorectal cancer, point-of-care screening, urine biomarkers, colorimetric biosensor, RGB sensor, metabolomics, low-resource settings

## Abstract

Early detection of colorectal cancer (CRC) is challenging in low-resource settings because access to colonoscopy and centralized laboratory testing is limited. Urine-based metabolite biomarkers offer a non-invasive alternative for CRC triage, but translating a laboratory assay into a point-of-care (PoC) system requires standardized fluid handling, operator-independent timing, field-compatible reagents, and quantitative calibration. Here, we present a low-cost, semi-automated PoC platform integrating a validated, sequential, three-metabolite CRC-biomarker assay with robotic fluid handling, a motorized chromatographic module, an optical reader, Bluetooth electronics, and tablet-guided control. The platform measures urinary diacetylspermine and hippuric acid, with creatinine serving as a normalization reference, and reports absolute quantitative concentrations. Automated dilution, tube positioning, timed incubation, controlled column elution, and software-guided transfer reduce operator-dependent variation. Using pooled urine samples spiked at clinically relevant concentrations, the creatinine assay showed a strong quadratic response (0–50 mM, R^2^ = 0.998), as did the diacetylspermine assay (0–4 μM, R^2^ = 0.979), while the hippuric acid assay showed a linear response of R^2^ = 0.989. The RGB sensor tracked the concentration-dependent trend observed with a laboratory microplate reader (R^2^ = 0.968–0.999). Reagents reformulated as lyophilized or pre-weighed, vacuum-sealed kits withstood accelerated heat and humidity (45 °C/70% RH) testing and international shipping to the pilot site very well. At approximately USD 490 for the complete instrument (including tablet) and roughly USD 7.65 in consumables per screening test, the platform offers a practical, quantitative, and portable route for decentralized CRC screening.

## 1. Introduction

Early and accurate cancer detection is key to effective cancer treatment. Detecting cancer at an early stage can reduce mortality and morbidity while substantially increasing the likelihood of a complete and durable cure [1]. Despite decades of medical advances, cancer remains one of the leading causes of morbidity and mortality worldwide [2,3] and patient outcomes still depend heavily on cancer staging at diagnosis.

Colorectal cancer (CRC) exemplifies this challenge. CRC is the third most common cancer and a major cause of global mortality, with over 930,000 deaths reported in 2020. CRC is projected to cause 1.6 million deaths per year by 2040 [4,5,6]. CRC mortality risk rises markedly with advancing disease, as five-year survival decreases from approximately 90% for stage I and 85% for stage II to 65% for stage III and only 10% for stage IV disease [7,8]. Traditional CRC screening and diagnostic methods include several well-established imaging approaches such as ultrasound, computed X-ray tomography, PET, and MRI. However, direct visualization via colonoscopy is considered the global standard for both CRC screening and surveillance. Unfortunately, the high cost, specialized equipment, and skilled personnel required make it, and most other imaging approaches, inaccessible to many patients in low- and middle-income countries (LMICs). Within resource-limited settings, even patients with rectal bleeding and other obvious clinical CRC indications almost never undergo colonoscopy or other image-based screening due to financial and logistical barriers [9]. To optimize the limited colonoscopy capacity and reduce unnecessary procedures in LMICs, there is a pressing need for simple, cost-effective, non-image-based screening triage tools to identify patients at the highest risk of CRC.

A number of non-image-based CRC screening tests are beginning to appear, with most being based on the detection of molecular biomarkers. These markers span a wide range of molecular classes, including genes, DNA- and RNA-based sequences, proteins and enzymes, lipids, small molecules (also referred to as metabolites), extracellular vesicles, and circulating tumor cells [10]). These CRC biomarkers can be detected in various tissues and body fluids, including blood, urine, saliva and stool [11]. No single biosample matrix has emerged as universally preferred, however stool-based tests are widely recommended and used for CRC screening. Unfortunately, due to challenges associated with acquiring and processing stool samples, stool-based biomarker tests have performed poorly in many populations, especially across LMICs [12,13,14]. In particular, the most useful stool tests such as stool DNA-FIT tests and stool RNA-FIT tests require advanced PCR instruments, centrifuges, robotic equipment, and individuals with high-end technical skills which are not routinely available in LMICs.

Biosensor systems, due to their low cost and technical simplicity, have gained considerable traction in medical testing due to their ability to detect molecular biomarkers with high sensitivity and specificity [3,15,16,17,18]. Biosensor-based colorimetric and lateral flow assays, in particular, have emerged as a practical screening solution for infectious disease testing in low-resource and LMIC settings [19,20]). Their compatibility with ambient-temperature storage, minimal equipment requirements, and straightforward visual or sensor-based readouts make them strong candidates for decentralized biochemical testing. Mobile and smartphone-linked optical sensing platforms have likewise broadened access to decentralized disease detection, although variability in ambient lighting, camera hardware, and device-to-device calibration remains a barrier to fully quantitative use in the field [21]. A key question is whether biosensor systems could be migrated from infectious disease testing to cancer screening. A secondary question is whether they could be adapted to perform quantitative measurements (which are often needed in cancer testing). While most low-cost PoC biosensors are qualitative in nature, quantitative PoC systems do exist, including reader-assisted lateral-flow, paper-based fluorescence, and sample-specific calibration platforms. However, these systems generally require an external reader, carefully controlled calibration, or computational correction to convert signal intensity into concentration [22,23,24]).

Because of the issues associated with stool tests for CRC screening, our group has sought to develop and validate low-cost, quantitative biosensor-based assays for screening CRC using urine. We recently discovered that two urinary metabolites, hippuric acid and diacetylspermine, exhibit excellent diagnostic yield [25]). We have also developed simple, lab-based colorimetric assays and a low-cost optical sensor that quantitatively measures these metabolites, together with a robust algorithm for CRC classification [25,26,27,28,29]). The analytical performance, specificity, and clinical relevance of these individual urinary assays in modern lab settings have been described in several previous publications [26,27]. Building on this work, we sought to determine whether the individual assays, an optical sensor, and a classification algorithm could be combined into a quantitative, field-ready CRC biosensor screening system suitable for deployment in low-resource settings.

Here, we describe the development of a low-cost, quantitative, semi-automated CRC screening system for field deployment in which the hardware and fluidic architecture are central components rather than simple accessories to the system’s optical sensor. The system mechanizes the most variable steps of the workflow: volumetric urine dilution, reagent and sample movement, tube indexing, controlled chromatographic elution, timed incubations, and synchronized optical measurement. These functions are coordinated by a tablet-based Android application that provides stepwise prompts, controls motors and pumps, records metadata, applies assay-specific calibration equations, and reports absolute analyte concentrations. By combining robotic assistance with pre-packaged reagents, the system was designed to reduce training requirements, improve reproducibility, and make multi-step metabolite testing practical outside a well-equipped clinical chemistry laboratory. As a final test, the CRC screening system and assay kits were sent to our collaborators in Nigeria, where they were successfully deployed in a pilot CRC screening study [26].

To our knowledge, this is among the first field-deployed, urine-based biosensor platforms to integrate quantitative measurement of multiple CRC-associated metabolites with semi-automated sample processing and concentration-based digital reporting. Collectively, this work establishes an affordable and practical platform for measuring CRC-associated urinary biomarkers in settings with limited laboratory infrastructure.

## 2. Materials and Methods

### 2.1. Overview of the Point-of-Care (PoC) Biosensor Workflow

**Figure 1:**
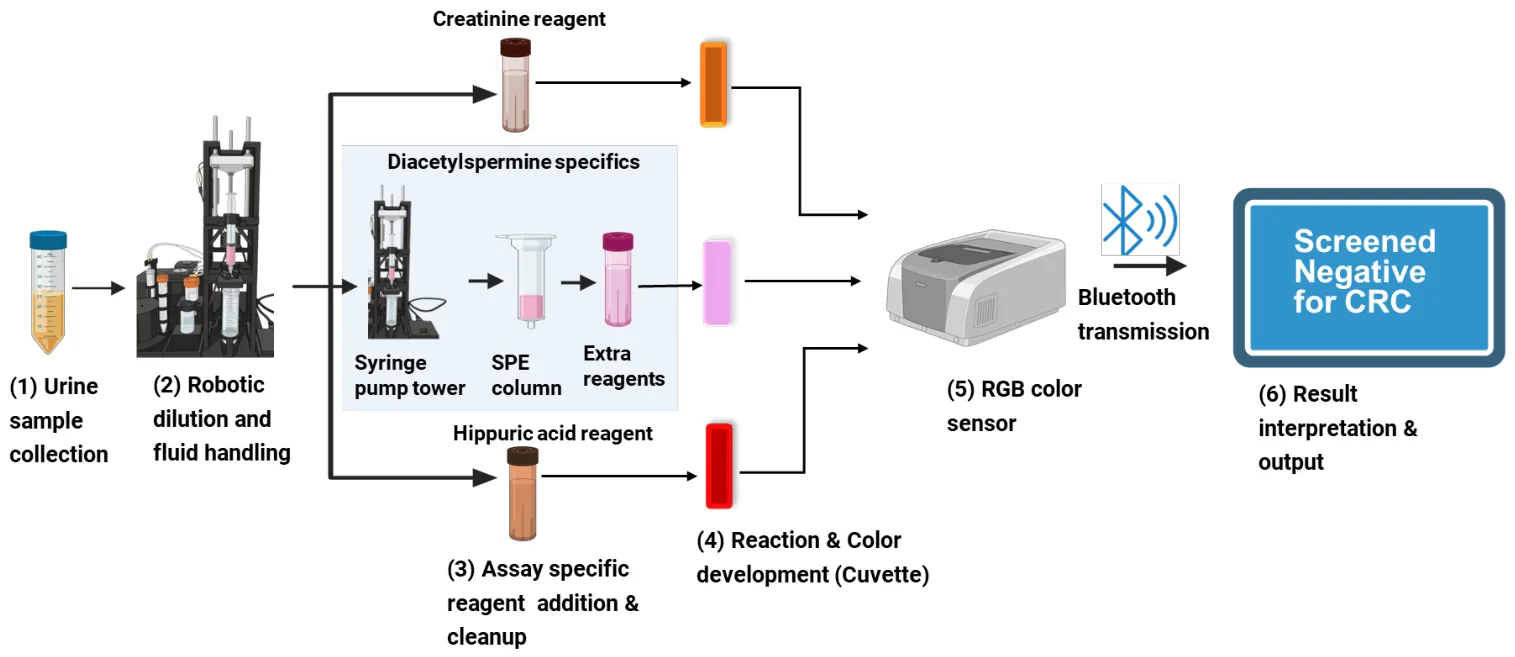
Integrated semi-automated workflow of the PoC biosensor platform, showing robotic dilution and fluid handling, assay-specific processing, quantitative RGB measurement, Bluetooth control, and concentration reporting.

Our PoC biosensor platform was designed to enable decentralized urine-biomarker testing using low-cost hardware, assay-specific colorimetric chemistry, and a simplified, software-guided workflow (Figure 1). The platform is comprised of four integrated modules: (i) a portable RGB optical sensor unit for colorimetric readout; (ii) an automated fluid management and measurement station incorporating peristaltic pumps and a tube-positioning carousel; (iii) a mechanized chromatographic tower for controlled elution and filtration for the diacetylspermine assay; and (iv) a Bluetooth-connected Android tablet running a custom robotic control and assay interpretation application.

The platform was configured to quantify the two urinary analytes relevant to CRC biomarker classification, diacetylspermine and hippuric acid, as well as a third metabolite, creatinine, which serves as a reference or concentration normalization analyte. These three analytes correspond to the previously developed CRC urine-biomarker panel used in our prior translational and pilot work [25].

The three colorimetric chemistries were adapted from previously developed laboratory assays for creatinine [30,31], for diacetylspermine [28], and for hippuric acid [29]. Similarly, the optical readout was based on the RGB sensor platform described in Bai et al. [27]. For field deployment, assay components were reformulated into on-site kits using pre-measured, freeze-dried, or otherwise field-compatible reagents wherever possible, and the original manually intensive workflow was automated or mechanized to minimize manual preparation while preserving assay performance. A comparison of the three field-adapted workflows is summarized in Table 1.

### 2.2. Preparation of the On-Site Creatinine Assay

The original laboratory creatinine assay was adapted for on-site use as a two-step colorimetric method consisting of a urine pre-dilution step followed by alkaline reaction with 3,5-dinitrobenzoic acid (DNBA). Under alkaline conditions, creatinine reacts with DNBA to form a purple creatinine–dinitrobenzoate complex, with color intensity increasing quantitatively as a function of creatinine concentration. Because urinary creatinine is present over a relatively broad physiological concentration range, a reproducible dilution step was required before color development. For the on-site format, this 20-fold dilution was mechanized using the automated fluid management station to reduce operator variability. Specifically, 100 µL of urine was diluted with 1900 µL of Milli-Q water before the reaction.

DNBA, lithium hydroxide monohydrate (LiOH·H_2_O), carboxymethylcellulose (CMC), and sucrose were purchased from Sigma-Aldrich (St. Louis, MO, USA), and all solutions were prepared using Milli-Q ultrapure water. A 0.5 mol L^−1^ LiOH working solution was prepared by dissolving 2.098 g of LiOH·H_2_O in 100 mL of water. A 0.25 mol L^−1^ LiOH solution was prepared by 1:1 dilution of the 0.5 mol L^−1^ LiOH solution with water. The DNBA reagent (0.3 mol L^−1^) was prepared by dissolving 0.0636 g of DNBA in 1 mL of 0.5 mol L^−1^ LiOH.

To create a field-stable reagent format, the liquid DNBA/LiOH reaction chemistry was reformulated as a lyophilized single-use reagent packaged into easy-to-use, easy-to-ship vials. The lyophilized formulation contained DNBA under alkaline LiOH conditions, with 10% (*w*/*v*) sucrose and 2% (*w*/*v*) CMC added as stabilizing excipients. Aliquots of 100 µL were dispensed into 0.5 mL cryogenic vials fitted with pierced caps to permit lyophilization of the frozen solutions to occur. The vials were frozen at −80 °C in the dark for 1 h to ensure uniform solidification and then lyophilized using a freeze-dryer (Labconco Corporation, Kansas City, MO, USA) with a primary drying program at a chamber temperature of −78 °C and a pressure of 0.091 mbar. After 16 h, fully dried vials were capped with standard non-pierced lids. To prevent premature color development and protect the reagent from atmospheric moisture, each vial was vacuum-sealed in a light-tight Mylar bag containing a silica-gel desiccant packet and an oxygen-absorber packet for facile transport and long-term storage.

For assay validation, commercial pooled urine (purchased from Medix Biochemica, Maryland Heights, MO, USA) was spiked with creatinine to generate calibration samples across the expected urinary concentration range found in humans, including 0.625, 1.25, 2.5, 5, 10, 15, 20, 25, 30, 40, and 50 mmol L^−1^. Each spiked urine sample was diluted 20-fold before analysis.

For assay validation with the on-site CRC screening system, a total volume of 100 µL, consisting of 50 µL of diluted creatinine-spiked urine, 25 µL of 0.3 mol L^−1^ DNBA, and 25 µL of 0.25 mol L^−1^ LiOH was mixed thoroughly, incubated at room temperature for 10 min, and then transferred to the device’s custom optical cuvette for RGB-based detection using the on-site CRC screening system. Parallel measurements were performed by measuring the absorbance at 490 nm with a standard microplate reader (Section 2.9).

### 2.3. Preparation of the On-Site Diacetylspermine Assay

Diacetylspermine, also referred to as N1, N12-diacetylspermine, is a low-abundance urinary polyamine metabolite associated with colorectal cancer and other malignancies. In contrast to creatinine, which is present in urine at millimolar concentrations, urinary diacetylspermine is present at substantially lower concentrations and therefore requires significant sample clean-up and enrichment before colorimetric detection. Amberlite IRA 400/402 anion-exchange resin, Amberlite CG120 II/Serdolit CG120 II cation-exchange resin, potassium chloride, sodium carbonate, sodium bicarbonate, sucrose, trehalose, and DMSO were purchased from Sigma-Aldrich (St. Louis, MO, USA). N1,N12-diacetylspermine HCl and Amplex Red/OxiRed were purchased from CaymanChemicals (Ann Arbor, MI, USA), and Horseradish peroxidase (HRP) was purchased from Thermo Fisher Scientific (Waltham, MA, USA). BD syringes were obtained from Becton, Dickinson and Company (Franklin Lakes, NJ, USA), and resin columns were assembled in-house.

The on-site diacetylspermine assay was adapted from our previously reported laboratory method [28]. It is based on two linked processes: selective enrichment of diacetylspermine from urine using solid-phase resin columns, followed by enzymatic color development. Detection and quantification is based on a coupled enzymatic–colorimetric reaction. A recombinant diacetylspermine oxidase (rDAS Ox), expressed and purified from *Escherichia coli*, oxidizes diacetylspermine and, among its products, generates hydrogen peroxide (H_2_O_2_) in an amount proportional to the diacetylspermine present. HRP then uses this H_2_O_2_ to oxidize a colorimetric peroxidase probe (OxiRed; the Amplex Red probe, 10-acetyl-3,7-dihydroxyphenoxazine) to the pink–red product resorufin. Because resorufin formation is stoichiometrically coupled to H_2_O_2_ generation, the intensity of the developed pink/red color is directly proportional to the urinary diacetylspermine concentration. As several endogenous urinary metabolites interfere with these oxidation reactions, the enzymatic step is preceded by a two-stage ion-exchange clean-up that removes interfering species and concentrates diacetylspermine (~15-fold) before color development.

For field deployment, the assay reagents are supplied as a pre-packaged, dried kit. The two ion-exchange resins are pre-loaded into single-use syringe columns (a 10 mL syringe for the first resin and a 1 mL syringe for the second) assembled with 3D-printed O-rings and connectors. The enzymes are supplied as a single light-protected dried reagent containing rDAS Ox (36 µg mL^−1^) and HRP (0.3 µg µL^−1^), and the chromogen is supplied as a separate light-protected dried aliquot (≈5 µg OxiRed per test); both are shielded from light to prevent premature oxidation. The reaction buffer is provided as a dried aliquot of carbonate–bicarbonate buffer (700 µL of 0.5 mol L^−1^ Na_2_CO_3_/NaHCO_3_) in a capped tube, which both receives the column eluate and provides the alkaline pH required for the enzymatic reaction. A potassium chloride elution solution (≈0.1 mol L^−1^) was prepared on first use by diluting a 2.5 mol L^−1^ KCl stock (400 µL made up to 10 mL with Milli-Q water).

In the original laboratory-based procedure, flow rate and elution efficiency through the resin steps depended heavily on operator skills and training. For the on-site CRC screening system, these steps were mechanized and automated using our system’s chromatographic elution tower (Section 2.5). When the diacetylspermine assay is run on the on-site CRC screening system, a 7 mL aliquot of undiluted urine is dispensed onto the assembled two-resin column and drawn through under controlled flow. The resin bed is then washed with 4 mL of water, excess water is removed, and the bound diacetylspermine is eluted with 400 µL of the KCl solution directly into the dried carbonate–bicarbonate buffer tube. The eluate is mixed and clarified through a 0.45 µm syringe filter, and 105 µL of the clear filtrate is used to reconstitute the dried enzyme reagent. This mixture is then transferred onto the dried chromogen, mixed, and a 100 µL portion dispensed into the custom optical cuvette for RGB-based detection after 15 min incubation at room temperature. The manual filtration, reconstitution, and dye-transfer steps are performed under timed prompts from the on-site CRC screening system’s tablet software to standardize timing and avoid operator inconsistencies. Because this assay involves the greatest number of operator-sensitive steps, it was supported by both software-guided instructions and mechanical assistance from the station.

### 2.4. Preparation of the On-Site Hippuric Acid Assay

Hippuric acid is a urinary metabolite included in the colorectal cancer-associated urine biomarker panel. The on-site hippuric acid assay was adapted from our previously reported colorimetric assay for quantifying hippuric acid in human urine [29]. Hippuric acid, pyridine, and p-toluenesulfonyl chloride (p-TsCl) were purchased from Sigma-Aldrich (Oakville, ON, Canada) and absolute ethanol was purchased from Greenfield Global (Brampton, ON, Canada).

The assay relies on the classical pyridine/arylsulfonyl chloride color reaction for detecting and quantifying hippuric acid [29]. To make the assay safer and more portable, the unstable, hazardous liquid benzene–sulfonyl chloride used in earlier methods was replaced with solid p-TsCl, which is much more stable. In this reaction, hippuric acid reacts with p-TsCl in the presence of pyridine to generate a colored product whose intensity is proportional to the hippuric acid concentration. The color-forming reaction is understood to proceed through sulfonyl chloride-promoted cyclization of hippuric acid to a reactive azlactone (2-phenyloxazol-5(4H)-one) intermediate, followed by condensation involving pyridine. In the present field-adapted format, this reaction is read as a concentration-dependent brown coloration suitable for RGB-based quantification. Several urinary hippuric acid analogues (e.g., methyl-, p-amino- and p-hydroxy-hippuric acid) can react with p-TsCl and pyridine in the same manner and contribute a small background signal, but they are present at much lower urinary concentrations than hippuric acid and do not materially affect quantification [29].

For the on-site CRC screening system, the reagent that most constrains the workflow is p-TsCl, which must be dissolved immediately before use because the resulting solution is only briefly stable. Accordingly, solid p-TsCl was pre-weighed into individual microcentrifuge tubes for facile shipping and sample preparation. When the assay is run the p-TsCl must be dissolved, on-site, in 500 µL of ethanol immediately prior to the reaction (the dissolved reagent remains usable for ~20 min). As part of the on-site CRC screening system, pyridine is supplied as a pre-measured stock solution in a glass vial and aliquoted for each test. Similarly, ethanol is supplied as a stock solution in 50 mL conical (Falcon) tubes.

Relative to the diacetylspermine assay, the hippuric acid workflow requires little robotic intervention. Indeed, the fluid management station is used mainly for standardized dilution, timer-guided handling prompts, and final measurement. When the on-site CRC screening system for hippuric acid is run, urine is first diluted with water using the station’s peristaltic pump (100 µL urine into 900 µL water). For each reaction, 100 µL of the diluted urine is combined with 20 µL of pyridine, followed by 30 µL of the freshly prepared p-TsCl/ethanol solution. The solution is then mixed thoroughly to dissolve any residual solid, and a 100 µL portion dispensed into a custom-designed optical cuvette and read on the RGB sensor after an 8 min incubation at room temperature. For validation, pooled urine spiked with known hippuric acid concentrations was processed and measured by the identical on-site protocol (Section 2.9).

### 2.5. Sensor Station Design and Fabrication

**Figure 2:**
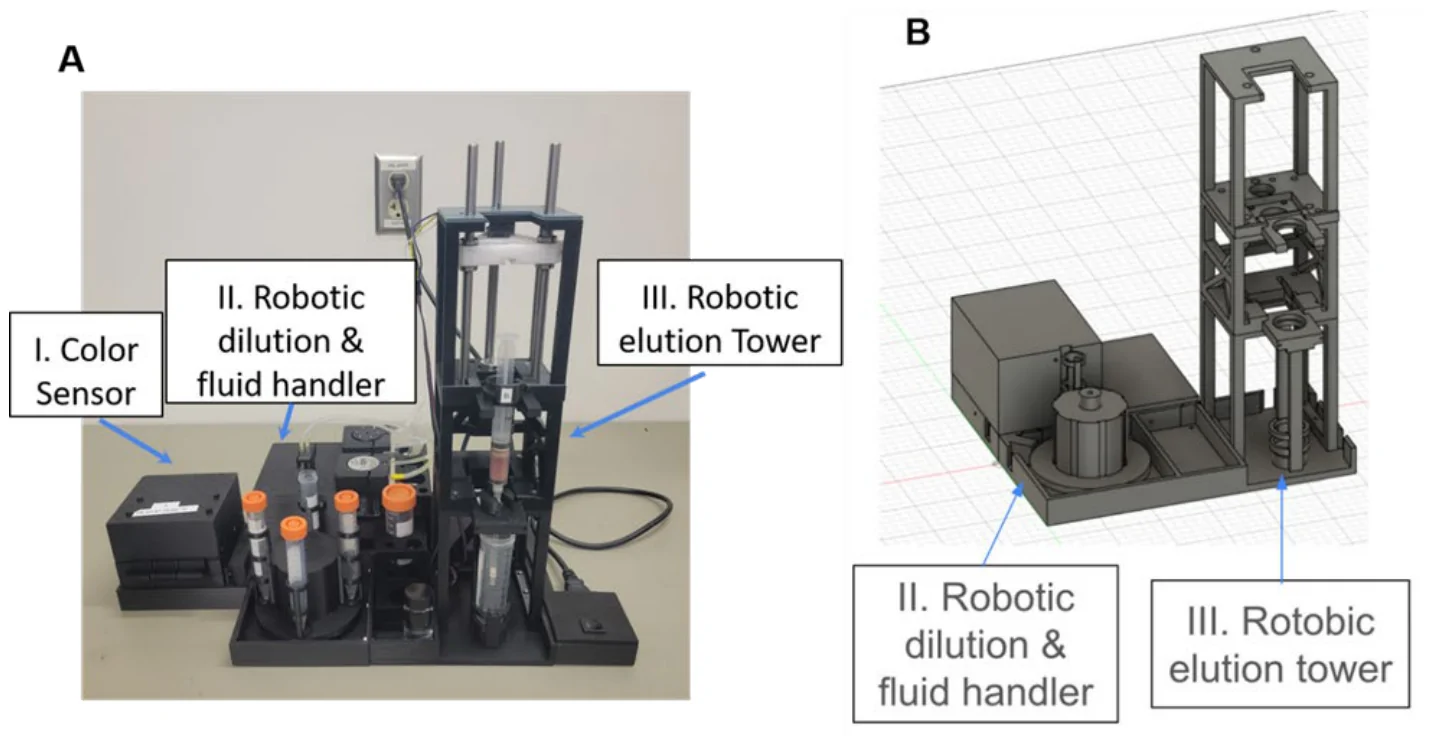
(**A**) Semi-automated CRC screening station comprising (I) the color sensor module, (II) the robotic dilution and fluid handler module with peristaltic pumps and motorized tube carousel, and (III) the robotic elution module for controlled syringe-column processing. (**B**) 3D render 3D printed components of the station.

The semi-automated CRC screening station is engineered as a modular benchtop robot, which standardizes sample processing while maintaining portability, repairability, and cost-effectiveness (Figure 2). It comprises three physical modules: (I) the color sensor module; (II) the robotic dilution and fluid handler module which includes peristaltic pumps and a motorized tube carousel; and (III) the robotic elution tower module, which controls syringe-column motion and elution. The modular layout permits each subsystem to be serviced or replaced independently. It also allows future assays to use the same control electronics, tablet interface, and optical reader.

The optical sensor unit used for our field-deployable, on-site CRC screening system resembled the low-cost RGB architecture of our previously reported portable colorimetric sensor [27]. It was modified with additional pin-outs to interface with the fluid management station. The sensor system houses three RGB color sensors and three white LEDs within a light-tight enclosure to minimize ambient interference. The system also holds the control board that coordinates the station and the Bluetooth module used to communicate with the tablet. During measurement, the LEDs illuminate the sample and the RGB sensors acquire the transmitted color response. The sensor body was 3D-printed in black polylactic acid (PLA) filament (Amazon, amazon.ca); the printed circuit (control) board was fabricated by PCBWay. The RGB sensor chip, Bluetooth module, LEDs, and passive components (resistors, capacitors) were obtained from Digi-Key.

The automated fluid management system is completely new for this on-site CRC screening system. It uses peristaltic pumps built from stepper motors and 3D-printed components, with plastic tubing for reproducible dispensing of water and urine. A rotating carousel holding four 15 mL tubes positions samples beneath each pump. The carousel position is driven by a stepper motor and indexed using a Hall-effect sensor and magnet. The chromatographic elution tower is also completely new and provides precise flow-rate control during the resin deposition, elution, and filtration steps of the diacetylspermine assay. This is done using a vertically driven lead-screw mechanism powered by a stepper motor with linear guides and bearings to ensure even mechanical force. Interchangeable adapters accommodate different syringe sizes and assay-specific processing modules.

All major structural components were 3D printed using PLA filament. Stepper motors for the pumps, carousel, and elution tower were obtained from StepperOnline (Wilmington, DE, USA); the limit switches, motor drivers, power board/switch, and other electronics from Digi-Key (Thief River Falls, MN, USA); and the metal support rods, lead-screw assembly, bearings, and tubing from McMaster-Carr (Elmhurst, IL, USA). Custom cuvettes were cut from layers of clear acrylic and bonded with clear epoxy (McMaster-Carr). To standardize sample handling, each kit also included fixed-volume pipettes (200 µL and 1 mL (IKEME fixed-volume pipettes (Guangzhou IKEME Technology Co., Ltd., Guangzhou, Guangdong, China) with matching tips (Axygen Inc., MilliporeSigma Canada Ltd. (Oakville, ON, Canada)); all assays were validated on these same pipette models. Most parts, including the peristaltic pumps and sensor/control unit, are fully swappable with simple ribbon cable connections.

### 2.6. Mobile Application and System Control

**Figure 3:**
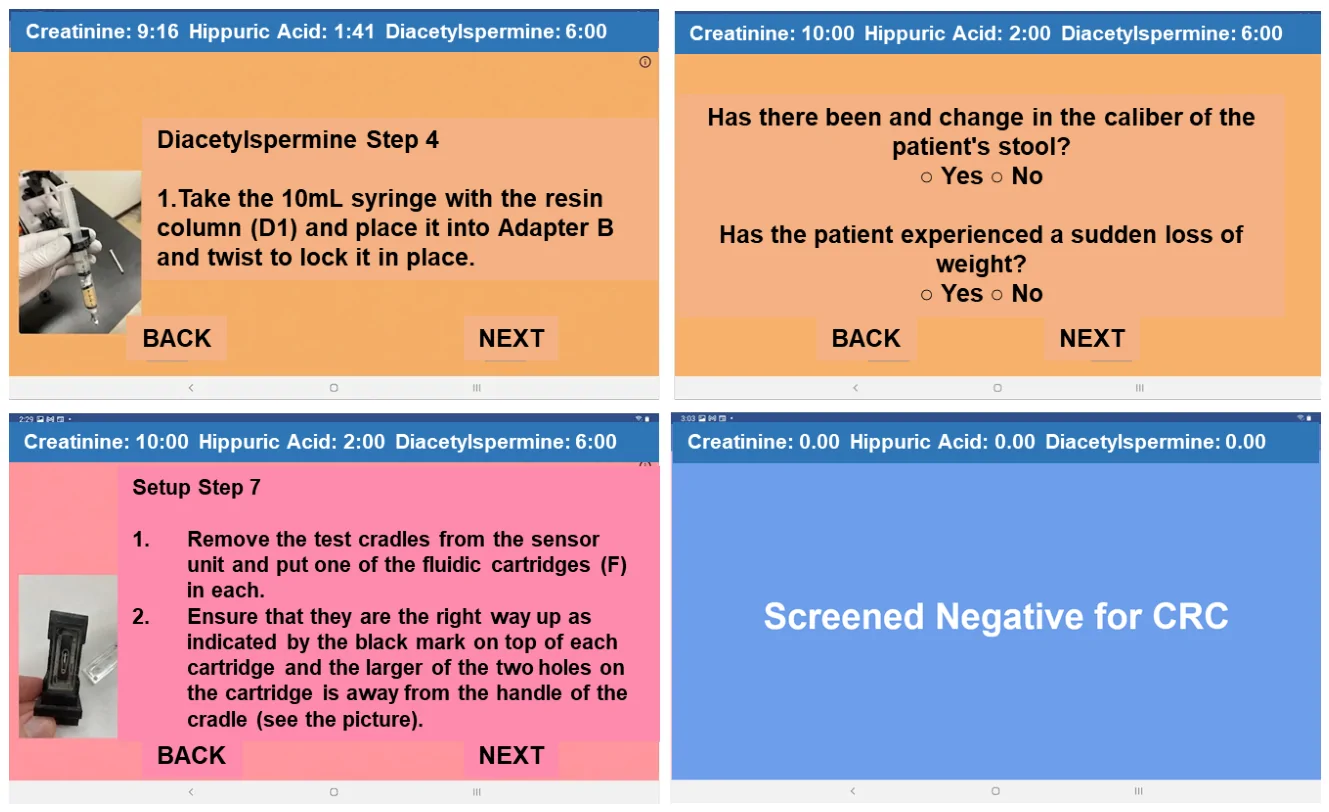
App interface workflow, screenshots of the sensor control app.

An Android application developed in Android Studio serves as the supervisory controller, user interface, and data-management layer for the semi-automated station. Running on a Samsung Galaxy Tab A7 (BestBuy, Edmonton, AB, Canada), the application communicates with the hardware by Bluetooth and issues commands to the pumps, carousel, chromatography drive, LEDs, and RGB sensors. Assay-specific workflows are encoded as ordered sequences of motor actions, timed pauses, prompts, and measurement steps. The application therefore does more than display instructions: it coordinates robotic operations, enforces incubation times, prevents steps from being performed out of sequence, captures patient and sample metadata, applies calibration equations, and reports absolute concentrations. This software-guided architecture reduces the amount of assay-specific training required and provides a traceable record of each measurement. Example interface screens are shown in Figure 3.

### 2.7. Optical Signal Acquisition and Color-Number Computation

Using commands from the Android application, the LED module illuminates the sample for a fixed duration (2 s) and the RGB sensors detects the transmitted light. The readings from the raw sensor channels are digitized, transmitted to the application, and normalized to relative intensities. Following the approach validated in [27], the color number is calculated using Equation (1), the color percentage calculated with Equation (2), and the color weight as calculated using Equation (3). The color percentage is calculated from color values which are the raw data obtained from sensor readings (either a red value, blue value, or green value to generate a red color percentage, blue color percentage, or green color percentage, respectively). The color weight is obtained using Equation (3), where the slope and correlation index (R^2^) values from the calibration curves for each color are inserted. Each color has its own weight specific to that range of colors or color reaction. The color number Equation (1) is a sum of all colors multiplied by their own color weight.

(1) $$Color\;number=⅀(\left(\frac{Color\;percent}{100}\right)\times color\;weight)$$

(2) $$Color\;percent=100\times (\frac{color\;value}{red\;value+green\;value+blue\;value})$$

(3) $$Color\;Weight=SIGN(slope)\times (\left({1,000,000}^{\left(\frac{\left|slope\right|}{100}\right)}-1\right)\times \left({1,000,000}^{R^{2\;}}\right))$$

The color number is a dimensionless composite value whose numerical scale is specific to each assay, since the channel weights are derived from the color produced by that particular reaction. Absolute color numbers are therefore not comparable between assays and are converted to concentration units through each assay’s own calibration before interpretation.

### 2.8. Calibration and Data Handling

Calibration curves were constructed by plotting color numbers against known analyte concentrations for spiked, pooled-urine standards spanning the physiologically relevant range of each biomarker. The curves were fitted with linear or quadratic regression models as appropriate. The fitted models are embedded in the platform’s Android application and are used to convert measured color numbers into analyte concentrations during routine operation. After all reactions are complete, the application displays the screening outcome, and the full results can be exported as a csv file. User-entered metadata (e.g., sample identifiers and patient-specific clinical parameters) are stored alongside the measurements to support multi-sample and longitudinal analyses.

The analytical output of the platform is an absolute concentration rather than a color score. For each assay, standards of known concentration define an assay-specific calibration function stored in the application. The software applies the appropriate linear or quadratic inverse model to each measured color number and computes creatinine in mmol L^−1^ and diacetylspermine and hippuric acid in µmol L^−1^, from which creatinine-normalized biomarker values are derived. These continuous values are passed to the CRC risk-classification algorithm together with the user-entered clinical parameters, and the resulting screening outcome is shown on the results screen. The individual analyte concentrations are retained and exported, together with the date and time of the test and the final outcome, in a csv file for review, record-keeping, and downstream analysis. Presenting a single outcome at the point of care, while preserving the underlying concentrations for export, was a deliberate design choice intended to give the assay operator an unambiguous result without requiring on-site interpretation of numerical values. Although the calibration is empirical and must be verified for each reagent lot and deployment environment, the platform’s output is therefore quantitative in concentration units even though the on-screen result is presented categorically.

### 2.9. Analytical Validation in Spiked Pooled Urine

Commercial pooled human urine (Medix Biochemica, Maryland Heights, MO, USA) was used for all analytical validation reported here. A single commercial pool was used deliberately, so that biological variability was held constant and hardware- and workflow-derived variability could be isolated; individual-sample performance in the target population is reported in the companion clinical pilot study [26]. Because a single matrix was used, the replicates described below are technical rather than biological.

For each assay, pooled urine was spiked with each analyte across the physiologically and clinically relevant range of that biomarker. The intrinsic creatinine concentration of the pool was determined by ^1^H NMR spectroscopy and used as the baseline when preparing the creatinine standards, so that each nominal calibrator concentration comprises the endogenous content plus the spiked amount; the same principle applies to the other two analytes, whose endogenous content is reflected in the non-zero calibration intercepts. Creatinine was spiked over 0–50 mM, encompassing normal urinary creatinine across the full range of hydration states, with most spot urines near 10 mM, the value conventionally used as the normalization reference. Diacetylspermine was spiked at 0, 0.5, 0.75, 1, 2, 3, and 4 µmol L^−1^; this range was selected against the population-specific distribution reported for the target cohort, in which more than half of Nigerian urines contained ≥1 µmol L^−1^ diacetylspermine by LC-MS/MS [25] rather than against generic reference intervals. Hippuric acid was spiked at 0, 50, 100, 200, 300, 400, and 600 µmol L^−1^ into ten-fold diluted pooled urine containing an endogenous hippurate background of approximately 950 µmol L^−1^ [29].

Each calibration level was measured with 3 technical replicates. Replicate preparations were made in independent batches; a dedicated inter-day precision study or inter-operator precision was not evaluated in this work. Limits of detection (LOD) and quantification (LOQ) were determined for each assay by the regression-based approach, LOD = 3.3 σ/S and LOQ = 10 σ/S, where σ is the standard deviation (SD) of the response and S the slope of the calibration curve. These limits characterize the colorimetric assay chemistries under the reaction conditions and timings adopted for field use, as determined by absorbance on the laboratory microplate reader. They are not intrinsic optical limits of the RGB color sensor, whose intrinsic read precision is high (CV 0.39–0.46% on fixed color standards; [27]); in each assay the limiting factor is the chemistry, reagent background, reaction kinetics, and, for diacetylspermine, the efficiency of the two-resin enrichment rather than the optical readout.

Parallel reference measurements were made by transferring an aliquot of each reaction to a 96-well plate and reading absorbance on a BioTek 800 TS absorbance microplate reader (BioTek Instruments, Winooski, VT, USA) at 490 nm (creatinine) or 570 nm (diacetylspermine and hippuric acid). The validation strategy follows two stages: the assay chemistries were validated by absorbance on the microplate reader, and equivalence was then assessed between that reader and the low-cost RGB sensor. Cross-platform comparisons plot sensor color number against microplate-reader absorbance and are fitted by ordinary least squares linear regression. The quantitative accuracy of each assay rests on its own calibration against gravimetrically prepared standards of known concentration in a matrix-realistic background, not on this cross-platform comparison, and the sensor was not compared against an orthogonal analytical technique.

## 3. Results and Discussion

### 3.1. Validation of On-Site Creatinine Assay

**Figure 4:**
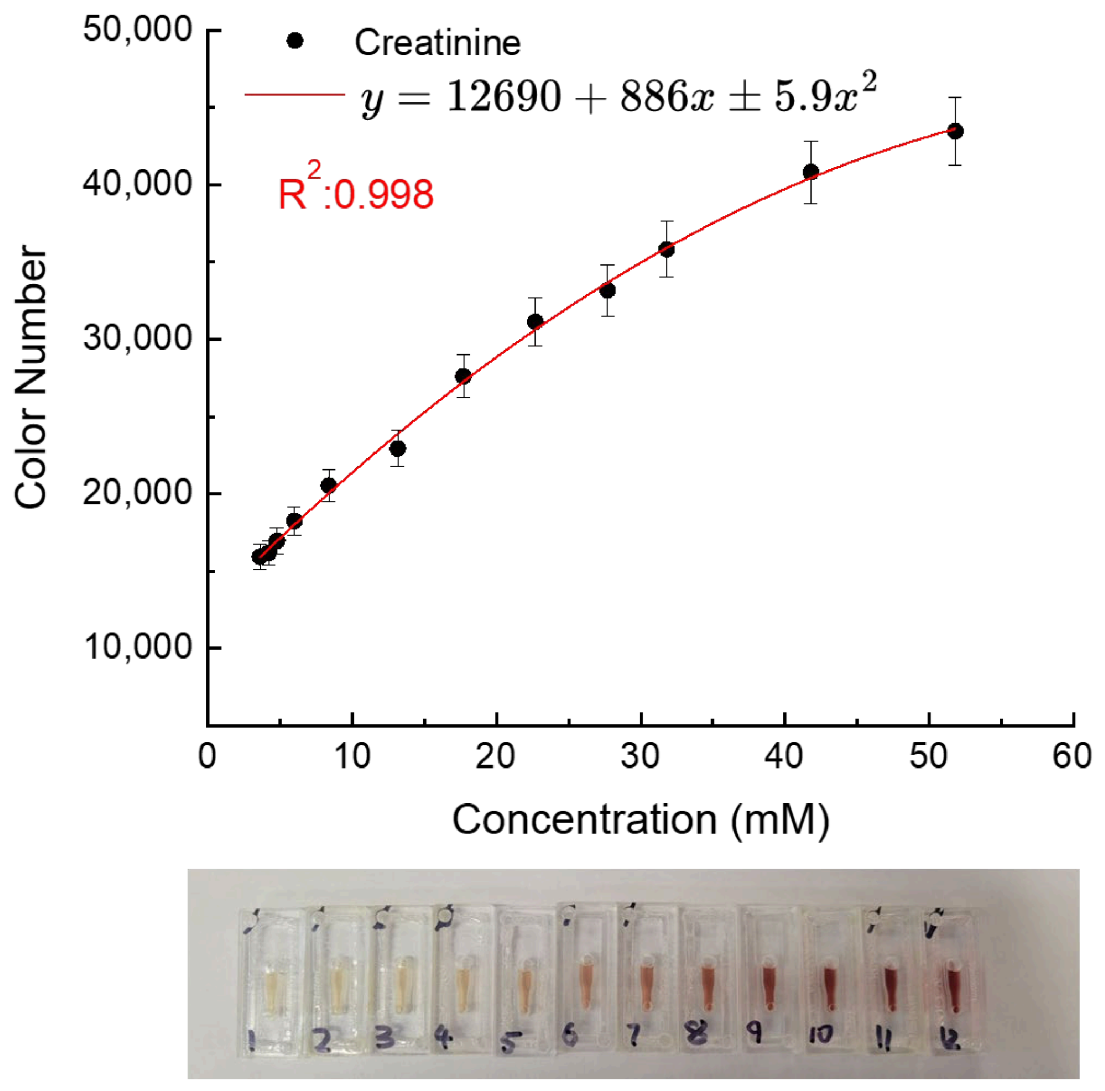
Creatinine calibration curve tested in pooled urine. The error bars represent the mean ± SD. A picture of the custom cuvettes with developed color is also shown; cuvettes 1–12 correspond to the twelve calibration standards, in order of increasing creatinine concentration. When the sensor-derived color number was plotted against the known creatinine concentration, the resulting calibration curve showed a strong quadratic relationship across the tested range (0–50 mM; R^2^ = 0.998). Because urinary creatinine concentrations can vary widely among individuals and clinical samples, the assay was intentionally validated over a concentration range extending beyond the normal physiological range to maximize the practical operating range of the CRC screening system’s RGB sensor. This behavior is consistent with our previous RGB sensor work [27], where creatinine quantification in urine also showed excellent calibration performance and slight nonlinearity at higher concentrations due to optical saturation effects at stronger color intensities. In that earlier work, the RGB color sensor yielded creatinine calibration performance comparable to a microplate reader, with high agreement across physiological urinary concentrations.

The results of measuring known spiked concentrations of creatinine in pooled urine using the CRC screening system’s RGB sensor are shown in Figure 4. Across the tested concentration range, the assay produced a clear concentration-dependent increase in developed color intensity. Visual inspection of the custom optical cuvettes confirmed a progressive, qualitative increase in yellow–purple coloration with increasing creatinine concentration.

In the present field-adapted version, the major translational changes included the use of DNBA, as well as dried or prepackaged reagents, automated sample dilution, and cuvette-based optical readout. Despite these modifications, the assay retained a nearly identical, strong, and predictable calibration response as reported previously. This is important because creatinine serves as a normalization anchor within the broader biomarker workflow.

### 3.2. Validation of On-Site Diacetylspermine Assay

**Figure 5:**
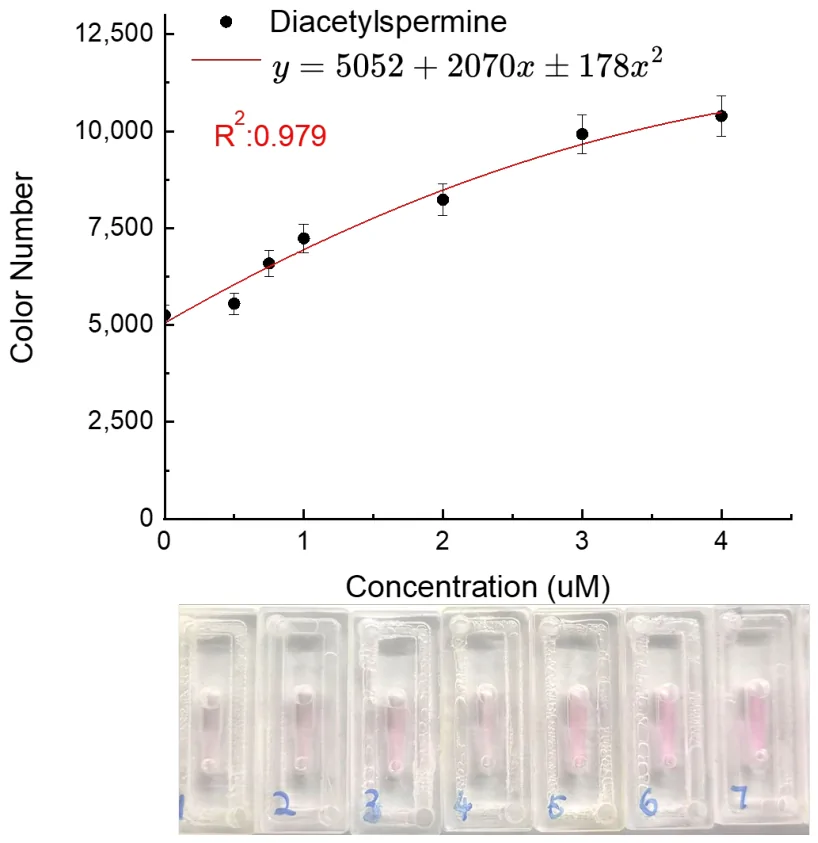
Diacetylspermine calibration curve tested in pooled urine. The error bars represent the mean ± SD. A picture of the custom cuvettes with developed color is also shown; cuvettes 1–7 correspond to the seven calibration standards, in order of increasing diacetylspermine concentration.

The results of measuring known spiked concentrations of diacetylspermine in pooled urine using the CRC screening system’s RGB sensor are shown in Figure 5 along with a best fit curve and a picture of the custom cuvettes showing the developed color.

Across the tested concentration range, a faint but concentration-dependent pink/red color developed, which was reproducibly detected by the RGB sensor. The resulting calibration curve showed a strong quadratic fit over the tested range (0–4 μM), with an R^2^ = 0.979. This indicates that the optical RGB sensor was sufficiently sensitive to quantify low-level analyte-associated color development despite the comparatively subtle visually detected signal. This result is particularly important from a translational perspective. Diacetylspermine is analytically more challenging than creatinine because of its lower concentration range and more complex chemistry, yet it is one of the most informative CRC-associated metabolites. The ability to integrate this assay into a portable field workflow supports the feasibility of moving beyond simple single-analyte strip tests toward more chemically sophisticated metabolite panels at the point of care.

### 3.3. Validation of On-Site Hippuric Acid Assay

**Figure 6:**
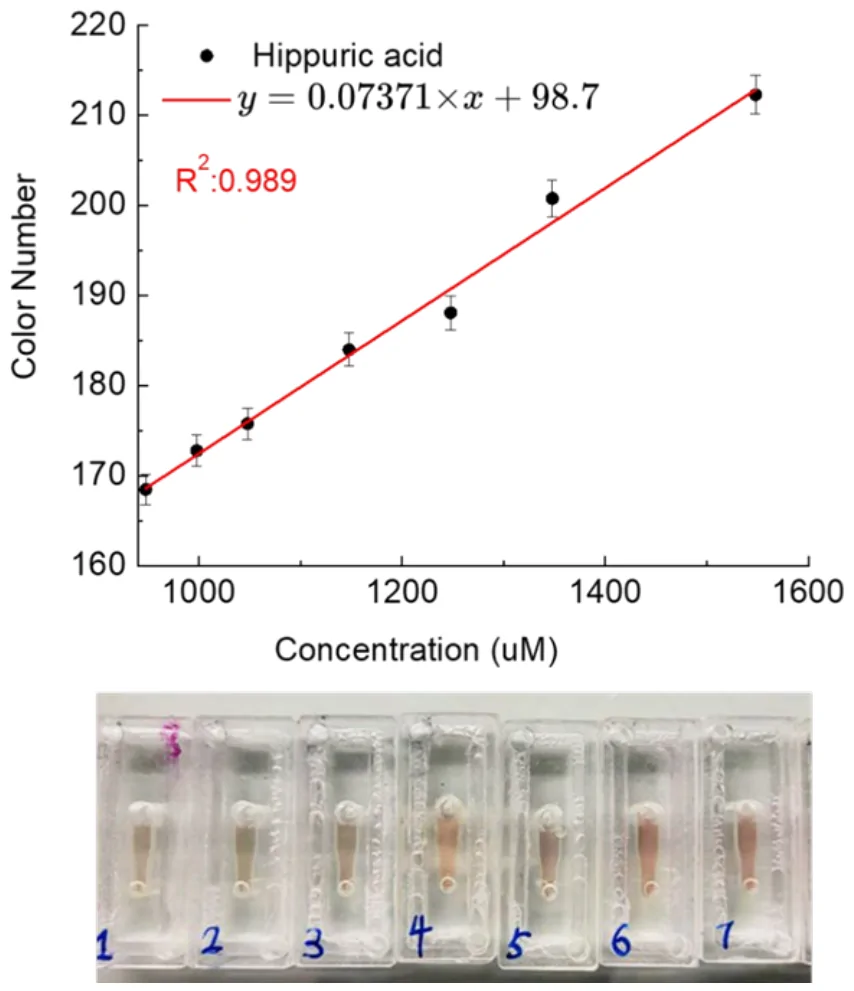
Hippuric acid calibration curve tested in pooled urine. The error bars represent the mean ± SD. A picture of the custom cuvettes with developed color is also shown; cuvettes 1–7 correspond to the seven calibration standards, in order of increasing hippuric acid concentration. Within the range of concentrations examined, the assay for quantifying hippuric acid resulted in a red–brown color that became more intense as the concentration of the analyte increased. This pattern was effectively captured by the RGB sensor, and the calibration curve derived from the data exhibited a strong linear correlation across the tested range with R^2^ = 0.989. The strong linearity demonstrates that field adaptation and reagent reformulation did not compromise quantitative performance. Although the visual response of the hippuric acid assay was less pronounced compared to the creatinine and diacetylspermine assays, it performed consistently in analytical terms, making it highly suitable for optical quantification through the color-number method.

The results of measuring known spiked concentrations of hippuric acid in pooled urine using the CRC screening system is shown in Figure 6 along with a best fit curve and a picture of the custom cuvettes showing the developed color. The hippuric acid assay remained relatively close to its original in-laboratory format, with the CRC screening system primarily being used for standardized dilution, timing support, and final measurement.

The preservation of linearity is advantageous for calibration simplicity and may facilitate more robust interpretation in future field use. In the broader CRC biomarker context, this result supports the feasibility of incorporating chemically diverse analytes into a single portable biosensing workflow.

The primary aim of these validation experiments was to assess if the field-adapted assays and the RGB sensor platform could consistently generate responses that vary with concentration across the necessary ranges for urinary CRC biomarker testing. Although the tests also established the sensor hardware’s absolute optical detection limits, this was not their primary focus. This distinction is crucial because the platform is intended for algorithmic CRC risk classification based on metabolite concentrations found in urine. Consequently, the assays were tested over ranges chosen to represent the anticipated physiological and clinically significant concentrations of each biomarker, covering the entire urinary creatinine range and the low-micromolar range pertinent to diacetylspermine.

For creatinine, the priority was to demonstrate excellent measurable and modelled response across the broad urinary concentration range required for normalization and downstream interpretation. For diacetylspermine, the key requirement was detection of a reliable signal over a narrow low-concentration range, where small changes may be relevant to the CRC classification algorithm. For hippuric acid, the assay was evaluated across a broader urinary concentration range relevant to expected biological variation. In all three cases, the CRC screening system detected reproducible changes in developed color across the selected ranges, with excellent calibration fits.

Table 2 summarizes the performance of the colorimetric urinary biomarker assays as implemented in the CRC screening system. The practical lower limits observed for these workflows were primarily governed by the color-forming chemistry and enzyme activity of the assays rather than by the RGB sensor hardware. For the reaction systems, the estimated LODs were 3.26 mM for creatinine, 0.08 µM for diacetylspermine and 75 µM for hippuric acid, with corresponding LOQs of 9.89 mM, 0.25 µM, and 226 µM. These data support the suitability of the platform for measuring biomarker concentrations within the ranges required for the CRC urine testing workflow. They also clarify that further improvements in low-end sensitivity would most likely require optimization of assay chemistry rather than changes to the sensor hardware.

### 3.4. Correlation with Microplate Reader Measurements

**Figure 7:**
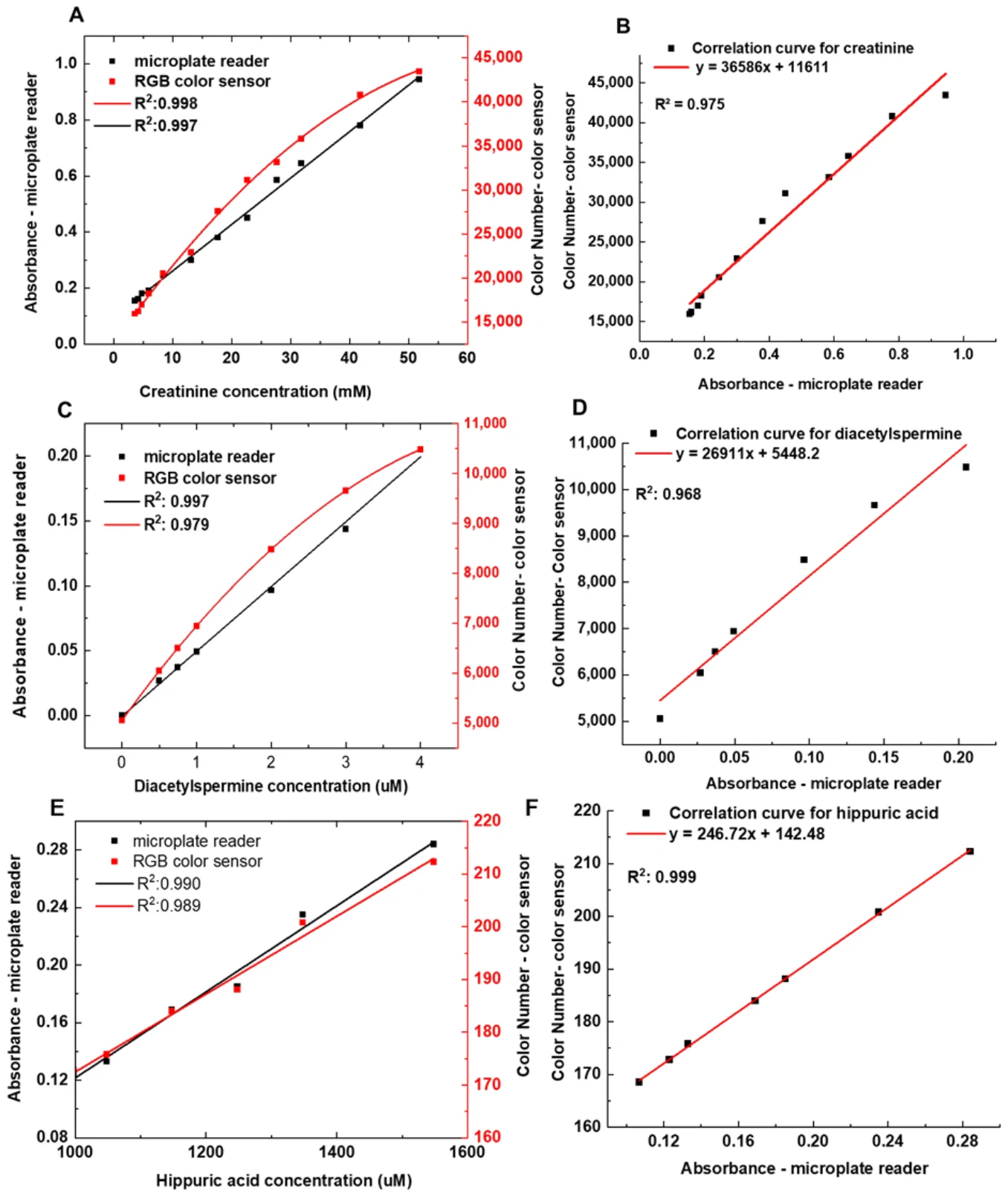
RGB color sensor versus microplate reader for creatinine (**A**,**B**), diacetylspermine (**C**,**D**), and hippuric acid (**E**,**F**). Panels (**A**,**C**,**E**) show concentration–response curves on both platforms for spiked pooled urine; panels (**B**,**D**,**F**) plot sensor color number against microplate-reader absorbance, fitted by ordinary least-squares linear regression. These cross-platform fits are empirical descriptions over the range tested and are not presented as evidence of equivalent analytical response. Absorbance was measured at 490 nm (creatinine) or 570 nm (diacetylspermine and hippuric acid).

To evaluate whether the portable RGB color sensor could reproduce measurements obtained using a microplate reader, sensor-derived color number values were compared directly with spectrophotometric absorbance measurements for creatinine, diacetylspermine, and hippuric acid. For each analyte, pooled urine samples spiked with known concentrations were analyzed using both the RGB sensor platform and a conventional microplate reader. The resulting concentration–response curves and cross-platform correlations are shown in Figure 7.

In the case of creatinine, both the microplate reader and the RGB sensor of the CRC screening system exhibited robust responses that varied with concentration across the range tested. The spectrophotometric calibration curve demonstrated an outstanding fit, achieving an R^2^ of 0.997, while the RGB sensor’s response was similarly well-aligned, with an R^2^ of 0.998. When comparing sensor color number values directly with spectrophotometric absorbance, a strong positive correlation was observed, with an R^2^ of 0.975. These findings suggest that the portable sensor effectively mirrored the overall analytical pattern observed with the microplate reader, even though it utilized RGB-based optical detection instead of wavelength-specific absorbance measurement.

The diacetylspermine assay produced comparable concentration–response behavior on both platforms. The microplate reader exhibited a pronounced increase in response proportional to concentration, achieving an R^2^ of 0.997. Similarly, the RGB sensor delivered a comparable outcome with an R^2^ of 0.979. When comparing the sensor to the microplate reader within the CRC screening system, an R^2^ of 0.968 was observed. Although this correlation was slightly less than that for other analytes, it is anticipated due to the lower concentration range of diacetylspermine, the subtle pink/red color change, and the more intricate sample preparation required for this assay. Notably, the RGB sensor successfully detected and quantified the color response associated with diacetylspermine, following the concentration-dependent response observed with the microplate reader.

For hippuric acid, both the microplate reader and the RGB sensor demonstrated highly consistent linear responses across the tested range. The calibration curve for the microplate reader exhibited an R^2^ value of 0.990, whereas the RGB sensor’s calibration curve showed an R^2^ value of 0.989. A direct comparison between the CRC screening system’s RGB sensor color number and spectrophotometric absorbance yielded an R^2^ of 0.999, indicating a closely linear relationship between the two readouts over the range tested. This close tracking suggests that the color-number approach employed by the portable sensor is particularly well-suited to the hippuric acid reaction, where the developed color increased proportionally with analyte concentration.

In summary, the three assays produced closely tracking responses on the RGB sensor of the CRC screening system and the microplate reader, with direct correlation R^2^ values of 0.975 for creatinine, 0.968 for diacetylspermine, and 0.999 for hippuric acid. These findings indicate that the RGB color sensor tracked the concentration-dependent trend observed with the microplate reader. The slightly lower correlation noted for diacetylspermine is likely due to the assay’s reduced signal intensity and increased workflow complexity, rather than any limitation of the sensor hardware itself. Collectively, these results support the RGB color sensor of the CRC screening system as a dependable and cost-effective optical readout for urinary biomarker testing in field settings.

The close tracking between the two readouts is important for translating these assays from laboratory workflows to decentralized PoC use. While microplate readers provide precise absorbance readings, they are generally less suitable in low-resource clinical settings because of their cost, size, power requirements, and need for trained operators. In contrast, the CRC screening system’s RGB sensor provides a compact and inexpensive alternative that can be integrated with automated sample handling and tablet-guided workflows. These observations indicate that laboratory-developed colorimetric assays can be transferred to a deployable biosensor format while preserving the concentration-dependent information available from the chemistry.

### 3.5. Reagent Stability and Field-Readiness

Because the CRC screening system is intended for deployment in hot, humid, low-resource settings (Nigeria), the field-adapted reagent kits were subjected to accelerated heat and humidity stress tests. These included multi-month controlled-temperature storage tests and round-trip international shipping tests. The complete dataset from these tests is provided in the Supplementary Information (Table S1 and Figures S1–S4) and summarized in Table 3. Across all three assays, the optimized packaging, where each dried component vacuum-sealed in a light-tight Mylar bag with a silica-gel desiccant and an oxygen absorber, preserved assay performance through these stresses.

The lyophilized creatinine reagent demonstrated stability for a minimum of 10 days without premature color development. The hippuric acid reagent set exhibited negligible changes after 7 days at 45 °C and 70% relative humidity. The vacuum-sealed diacetylspermine reagent maintained its activity for approximately 2 to 3 months under room-temperature, as well as under refrigerated, and frozen storage conditions. It also withstood round-trip shipping to Nigeria.

However, three operational considerations were identified. Diacetylspermine proved to be the most environmentally sensitive component, losing enzyme activity under prolonged (16 days) extreme conditions (45 °C and 70% relative humidity) even when sealed. The diacetylspermine standard degraded, although the enzymatic reaction itself remained valid. Therefore, we learned that it is advisable to confirm or refresh the on-site standard. Finally, a minor p-TsCl-related color shift in the hippuric acid assay supports generating a region-specific calibration curve under local conditions, and saturation of the silica-gel desiccant provides a simple visual check for moisture ingress.

**Figure 8:**
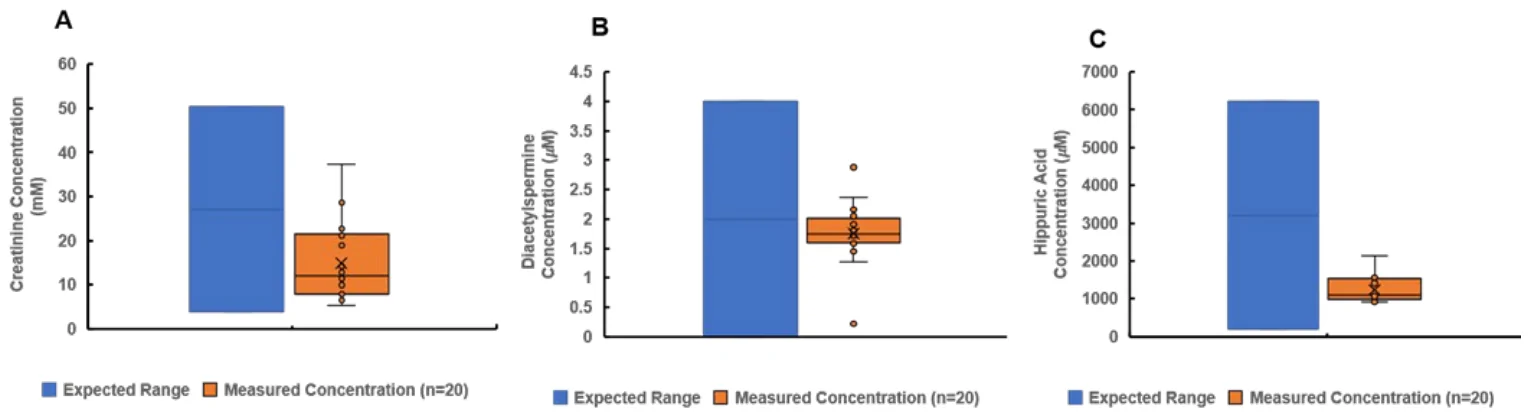
Biomarker concentrations measured on the portable platform in individual clinical urine specimens (*n* = 20) collected during the pilot deployment in Nigeria: (**A**) creatinine, (**B**) diacetylspermine, and (**C**) hippuric acid. Each point represents one specimen. Each plot is accompanied by the expected concentration range for each biomarker based on previous assessment of the Nigerian cohort samples. Screening outcomes and clinical performance for this cohort are reported separately [26]).

In addition to the analyses of the spiked pooled urine samples, we also analyzed individual clinical urine samples at the pilot deployment site (Figure 8). The measured biomarker concentrations in the individual clinical urine samples were within the anticipated and measurable concentration ranges: creatinine 5.3–37.2 mM (median 12.1 mM); diacetylspermine 0.21–2.88 µM (median 1.75 µM); hippuric acid 909–2147 µM (median 1111 µM). All three analytes were quantified above their respective limits of quantification in every specimen. Likewise, the measured values are consistent with reported urinary concentrations for these metabolites, with creatinine falling within the normal interval of 3.8–50.3 mM, hippuric acid within the normal interval of 190–6220 µM, and diacetylspermine with the expected concentration of ≥1 µM by LC-MS/MS analysis [25,28]. These results demonstrate that the platform returns absolute concentrations in authentic clinical specimens, under field conditions, across the range in which the CRC risk-classification algorithm operates.

The platform was deployed for field use at pilot study sites in Nigeria; the clinical findings themselves are reported separately [26]. We note that, for optimal clinical use, a region-specific calibration should be generated using locally stored reagent lots prior to clinical deployment to any new locations. Due to infrastructure constraints, comparison of the metabolite concentrations as measured by the RGB platform and by LC-MS-based laboratory reference assays on the corresponding specimens from the Nigerian participants was not performed. Ideally this should be a goal for future studies.

### 3.6. Comparison with Existing Point-of-Care Approaches, System Cost, and Limitations

These results can be placed in the context of existing CRC screening and biosensing approaches. Stool-based tests such as the fecal immunochemical test (FIT) are the most widely deployed non-invasive options, but they have shown variable performance across many low- and middle-income populations and depend on stool collection, which can limit acceptability, participation and adherence [12,13]. A urine-based CRC test is fully non-invasive and may improve participation in screening programs. Relative to other reported colorimetric and paper-based cancer biosensors, which are typically single-analyte and validated under laboratory conditions [11], the present platform measures a chemically diverse, three-analyte panel and has been thoroughly tested for field transport and storage. It also improves upon smartphone-camera colorimetric readouts, whose quantitative accuracy is sensitive to ambient lighting and device-to-device camera variation. In our case, a dedicated RGB sensor with on-board white-LED illumination inside a light-tight enclosure standardizes the optical measurement. Electrochemical biosensors for colorectal cancer can offer higher intrinsic sensitivity [15,17,18], but they generally require electrode fabrication and more complex instrumentation. On the other hand, the colorimetric approach used here prioritizes low cost, reagent stability, and operational simplicity for decentralized use.

The comparison with the microplate reader evaluates the optical readout stage rather than the analytical method as a whole. In particular, it establishes that the low-cost RGB readout preserves the concentration-dependent information available from the chemistry, but it does not constitute validation against an orthogonal analytical technique. A direct comparison of platform-derived concentrations against a reference method such as NMR or mass spectrometry on identical specimens was not performed and remains an important next step.

The CRC screening platform was fabricated almost entirely in-house from easily available components, keeping material costs low. Excluding design and labor, the sensor station cost approximately USD 310 to assemble; adding the Android tablet used as the user interface brings the complete instrument to approximately USD 490. The reagents and consumables required each CRC screening test amounted to approximately USD 7.65. A primary design mandate from the funding agency (the NIH) was to ensure that the total cost per test (including both consumables and the amortized instrument cost over the device’s service life) does not exceed USD 15. Because the amortized hardware contribution shrinks rapidly with use, this target is met once a device performs approximately 67 tests. The cost per test falls to approximately USD 12.55 at 100 tests, USD 10.10 at 200 tests, and USD 8.14 at 1000 tests or more, approaching the USD 7.65 consumable floor. The station is built for easy maintenance in the field, with parts such as the peristaltic pump tubing being cheap to replace. This should allow for a service life of hundreds to thousands of tests, keeping the cost per test within the target throughout the device’s lifespan.

An additional distinguishing feature of this biosensor system is the use of absolute, concentration-unit quantification across a three-analyte panel measured sequentially on shared hardware. Many PoC sensors such colorimetric strips and lateral-flow devices are optimized for binary or semi-quantitative decisions, whereas fully quantitative PoC assays generally require a reader and calibration strategy [23,24]. Examples of quantitative biosensors include sample-specific calibrated cell-free biosensors and reader-assisted fluorescence assays for multiplexed cardiac biomarkers [22,23]. The present system differs from all other quantitative PoC sensors in that it combines three distinct clinical chemistry assays, automated sample preparation, concentration-unit calibration, and assay normalization in one field-deployable workflow. This enables the CRC classifier to use continuous biomarker values and preserves vital information that would be lost in a yes/no readout.

Several limitations of this work need to be acknowledged. The practical lower limits of the diacetylspermine and hippuric acid workflows are predominantly determined by the color-forming chemistry and enzyme activity, rather than the sensor hardware. Consequently, improvements in low-end sensitivity are expected to arise primarily from the optimization of the assay chemistry, rather than enhancements to the optical system. Likewise, the system is not yet fully automated and so the aspirational goal of invariant operator function has not been achieved. A formal multi-operator comparison of the original manual analysis versus the semi-automated analysis on this platform (across all three assays) is planned for the next deployment stage. Likewise, because the system is a laboratory-made prototype, the true costs of having a mass-produced device fabricated via injection molding, professionally assembled, and fully certified as a medical testing device has not been determined.

## 4. Conclusions

We have described the development and analytical validation of a low-cost, semi-automated CRC screening platform for the absolute quantification of urinary biomarkers. The primary advancement lies not solely in the development of a low-cost, highly sensitive RGB sensor, but in the integration of assay chemistry with automated dilution, motorized tube positioning, controlled chromatographic processing, dual-source power, software-enforced timing, concentration calibration, and digital reporting. These components transform a set of labor-intensive, skill-sensitive laboratory assays into guided workflows, enabling execution with minimal infrastructure and reduced reliance on operators.

The integrated system produces reproducible concentration-dependent responses for all three analytes and reports results in absolute concentration units (µM or mM). The robotic and semi-automated functions standardize the most error-prone fluidic steps, while the dedicated optical enclosure avoid the lighting and camera variability associated with smartphone-only readouts. With a cost of about USD 490 for the instrument, including a computer tablet, and around USD 7.65 for consumables per CRC screening test, the cost per test drops below the USD 15 target after just 67 tests and nears the USD 7.65 consumable cost over its expected service life. However, manufacturing, quality assurance, and regulatory compliance costs will ultimately need to be incorporated into the full cost model. Reagent reformulation and stress testing confirmed suitability for hot, humid conditions.

Together, these results establish a practical bridge between the discovery of urinary biomarkers for CRC [25] and real-world PoC implementation [26]. More broadly, the results demonstrate that portable RGB-based colorimetric biosensing can provide a flexible, scalable framework for metabolite-based screening in settings where access to centralized laboratory infrastructure is limited.

Building on this device validation and pilot implementation, future work will focus on three main directions: expanding CRC screening in Nigeria beyond the pilot phase, developing additional colorimetric assays for new biosensor applications on the existing platform, and creating a fully automated version of the CRC screening system that requires minimal setup and no user intervention in field settings.

## Figures and Tables

**Table 1:** Comparison of the three field-adapted colorimetric assay workflows (creatinine, diacetylspermine, and hippuric acid), summarizing sample volume, on-station dilution, preparation complexity, incubation conditions, developed color, number of manual operator steps, and total assay time. RT, room temperature.

Step	Creatinine	Diacetylspermine	Hippuric Acid
Sample Vol.	100 µL urine	7 mL urine	100 µL
Dilution	Auto (peristaltic, 1:20)	Pump measure (no dilution)	Auto (peristaltic, 1:10)
Prep Complexity	Low (1-step lyophilized)	High (resins + syringe pump)	Medium (fresh TsCl mix)
Incubation	10 min @ RT	15 min @ RT	8 min @ RT
Color Developed	Yellow-purple	Pink/red	Red-brown
Operator Steps	3	7	5
Total Time	20 min	35 min	18 min

**Table 2:** Summary of the colorimetric urinary biomarker assays calibration implemented in the portable sensor platform.

Biomarker	Conc. Range	Fit Type	R^2^	Workflow LOQ *	Workflow LOD *	Physiological/Clinically Relevant Urinary Range
Creatinine	0–50 mM	Quadratic	0.998	9.89 mM	3.26 mM	3.8–50.3 mM [27]
Diacetylspermine	0–4 μM	Quadratic	0.979	0.25 μM	0.08 µM	>52% of Nigerian urines ≥1 µM; generic reference ~0.007 µM (normal), ~0.27 µM (disease) [25,28]
Hippuric acid	0–1550 μM	Linear	0.989	226 μM	75 µM	190–6220 µM [29]

* LOD and LOQ values characterize the colorimetric assay chemistries under field-adapted reaction conditions as determined on a laboratory microplate reader, and are not intrinsic optical limits of the RGB sensor.

**Table 3:** Summary of reagent stability and field-readiness for the three field-adapted assays (full data in Supplementary Information, Table S1).

Assay	Accelerated Stress (45 °C/70% RH)	Vacuum-Sealed Storage	Shipping and Field Guidance
Creatinine	Stable when vacuum-sealed; premature browning only if inadequately protected	Lyophilized reagent stable ≥ 10 days (Ab490 ≈ 0.4)	Original packaging failed in transit; resolved by vacuum-sealed Mylar with desiccant
Hippuric acid	Color formation never inhibited after 7 days; minor p-TsCl shift only at 10× dilution	Reagent set robust (lyophilized standard, pyridine, pre-weighed p-TsCl, ethanol)	Performed well round-trip to Nigeria; region-specific calibration advised
Diacetylspermine	Vacuum-sealed reagent survives short exposure; activity lost by 16 days; non-sealed reagent fails	Stable ~2–3 months at RT, 4 °C, and −20 °C	Reaction valid after shipping but standard degraded

## Data Availability

Data will be made available on request.

## Supplementary Materials

The following supporting information can be downloaded at: https://www.mdpi.com/article/10.3390/bios16090485/s1, Figure S1: Evolution of the assay-kit packaging. (A) Initial design using regular vacuum sealing around the dried reagent vials. (B) Optimized design: each dried component is vacuum-sealed (heat sealer, left) inside a light-tight Mylar bag containing a silica-gel desiccant packet and an oxygen-absorber packet. The optimized configuration prevented the moisture-driven premature color development seen with the initial design; Figure S2: Creatinine reagent stability. (A) Background absorbance (Ab490) of the lyophilized reagent versus days in storage; one inadequately protected condition (upper trace) developed premature color, while vacuum-sealed conditions remained low and flat. (B) Microplate time-course (days 1, 3, 7 and 10; five treatments) showing accelerated color development under heat and humidity. (C) Representative reconstituted reagent discs: left, spoiled (dark brown); right, intact (pale yellow); Figure S3: Hippuric acid reagent stability after 7 days at 45 °C/70% RH. (A) Undiluted standards in water (left rack) and urine (right rack) using fresh (G), non-vacuum-sealed stressed (S), and vacuum-sealed stressed (VS) p-TsCl; color is indistinguishable across conditions. (B) 10× diluted standards in water and urine, where a slight color difference between fresh and stressed p-TsCl is visible. (C) Independent repeats (two operators) using reagents shipped from Nigeria, across blank (0), concentrated, and 10× diluted samples in water and urine; Figure S4: Diacetylspermine (DAS) reagent stability. (A) Vacuum-sealed reagent stored at room temperature (~84 days), 4 °C and −20 °C (~68 days), with sucrose and trehalose stabilizers; the pink reaction color is retained. (B) Accelerated stress (45 °C/70% RH, 8 days): the vacuum-sealed batch sent to Nigeria (right, pink) survived, whereas a non-vacuum-sealed batch (left) failed. (C) Effect of international shipping on the diacetylspermine standard: a standard held in Edmonton (left) versus the same standard returned from New York (right); the latter is fainter (OD570 0.242 vs. 0.416); Table S1: Comprehensive summary of diacetylspermine assay stability across storage, shipping, and accelerated-stress conditions. All conditions used the optimized vacuum-sealed Mylar packaging with desiccant and oxygen absorber; Figure S5: Automated elusion system tested by NMR.
