# Supplementary material for: A Portable Colorimetric Biosensor Platform for Urinary Colorectal Cancer Biomarker Testing in Low-Resource Settings
Source: Biosensors (Basel). 2026 Sep 2;16(9):485. doi: 10.3390/bios16090485 (PMC13604405; doi:10.3390/bios16090485)
Supplement: Supplementary file 1 [file biosensors-16-00485-s001.zip › biosensors-4500087-supplementary.pdf]

Supplementary Information

A Portable Colorimetric Biosensor Platform for Urinary Colorectal Cancer Biomarker Testing in Low-Resource Settings

Prashanthi Kovur[#a], Scott MacKay[#a], Songtian Bai[b], James Cook[a], Claudia Torres-Calzada[a], Dipanjan Bhattacharyya[a], Upasana Singh[a], David S. Wishart[a,c-f]

[a] Department of Biological Sciences, University of Alberta, Edmonton, AB, Canada T6G 2E9.

[b] Department of Electrical and Computer Engineering, University of Alberta, Edmonton, AB, T6G 2H5, Canada

[c] Department of Computing Science, University of Alberta, Edmonton, AB, Canada T6G 2E8

[d] Department of Laboratory Medicine and Pathology, University of Alberta, Edmonton, AB, Canada T6G 2B7

[e] Faculty of Pharmacy and Pharmaceutical Sciences, University of Alberta, Edmonton, AB, Canada T6G 2H7

[f] Women and Children's Health Research Institute, University of Alberta, Edmonton, AB, Canada T6G 1C9

# These authors contributed equally

Corresponding author:
Dr. David S. Wishart
Dept. of Biological Sciences
CW-405, Biological Sciences Building
University of Alberta, Edmonton, AB, Canada, T6G 2E9
Telephone: 1-780-492 8574
Email: dwishart@ualberta.ca

S 1.1. Reagent reformulation and packaging

Liquid laboratory reagents were transformed into field-stable formats. The creatinine reaction mixture was reformulated into a single-step lyophilized reagent utilizing sucrose and carboxymethylcellulose as stabilizers. The diacetylspermine enzymes and chromogen were co-dried with a stabilizer, either sucrose or trehalose, and the clean-up resins were pre-packaged in syringes. Additionally, the hippuric acid reagents were provided as a lyophilized standard, pre-weighed p-toluenesulfonyl chloride (p-TsCl), and stock solutions of pyridine and ethanol. Two packaging configurations were evaluated (Figure S1): an initial design employing bubble-wrap pouches and an optimized design where each dried component was vacuum-sealed within a light-tight Mylar bag containing a silica-gel desiccant packet and an oxygen-absorber packet.

Initial field trials identified moisture exposure and premature color development as primary failure modes. With the initial packaging, both the creatinine and diacetylspermine reactions deteriorated during transit, and the silica-gel desiccant returned from the field was fully saturated. Transitioning to the optimized configuration, which involved vacuum-sealed, light-tight Mylar bags with desiccant and an oxygen absorber (Figure S1), resolved these issues and it became the standard packaging for all three assays. Vacuum sealing was particularly crucial for the moisture- and oxygen-sensitive components and was fundamental to the stability results presented below.

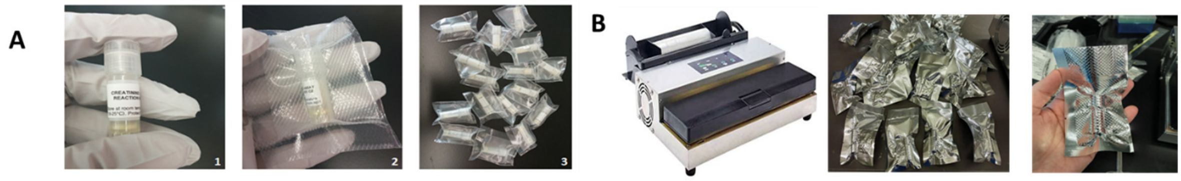

Figure S1. *Evolution of the assay kit packaging. (A) Initial design using regular vacuum sealing around the dried reagent vials. (B) Optimized design: each dried component is vacuum-sealed (heat sealer, left) inside a light-tight Mylar bag containing a silica-gel desiccant packet and an oxygen-absorber packet. The optimized configuration prevented the moisture-driven premature color development seen with the initial design.*

S 1. 2. Stress and stability testing

Accelerated stress testing was performed by holding packaged reagents in a controlled incubator at 45 °C and 70% RH conditions, chosen to approximate worst-case exposure during transit and at the pilot site. Hippuric acid reagents were stressed for 7 continuous days; diacetylspermine

reagents were evaluated at 8 and 16 days. Controlled-temperature storage stability was assessed for vacuum-sealed reagents held at room temperature (Edmonton, ~20–30 °C), 4 °C, and −20 °C for 68–84 days. In parallel, real-world stability was evaluated on kits that were shipped to Nigeria (round trip, ~4 months, ~25–45 °C and ~30–80% RH) and to a collaborator in New York, then returned and re-tested. For diacetylspermine, freshly prepared (non-vacuum-sealed) reagent batches were compared directly against the vacuum-sealed batch that had been sent to Nigeria.

Stability was assessed by running the standard colorimetric reaction after each stress or storage interval and comparing color development against freshly prepared reagents. Reactions were evaluated visually in the custom optical cuvettes or reaction tubes and, where indicated, quantified by absorbance on a benchtop plate reader (creatinine at 490 nm; diacetylspermine/chromogen at 570 nm). Hippuric acid stress tests were run with both undiluted and 10× diluted spiked standards (final 5 mM of hippuric acid) in water and in pooled urine using fresh p-TsCl (G), non-vacuum-sealed stressed p-TsCl (S), and vacuum-sealed stressed p-TsCl (VS). Saturation of the silica-gel desiccant (a color change from orange to green) was used as an indicator of moisture ingress during transport.

S 1.2.1 Creatinine reagent stability

The lyophilized creatinine reagent demonstrated stability when vacuum-sealed. Under most packaging and storage conditions, the background signal remained low and essentially constant ($Ab490 \approx 0.35$–0.46) for at least 10 days. However, a single condition lacking adequate protection exhibited progressive premature color development, with Ab490 increasing from approximately 0.38 to 1.0 over the same period (Figure S2A). Exposure to accelerated heat and humidity further expedited this browning process over time (Figure S2B). Spoiled reagent was easily distinguishable from intact reagent by its dark brown color, as opposed to the expected pale yellow (Figure S2C). These findings confirm that the failure mode involves premature, moisture-driven color development rather than a loss of reactivity, and that this issue is mitigated by optimized vacuum-sealed packaging.

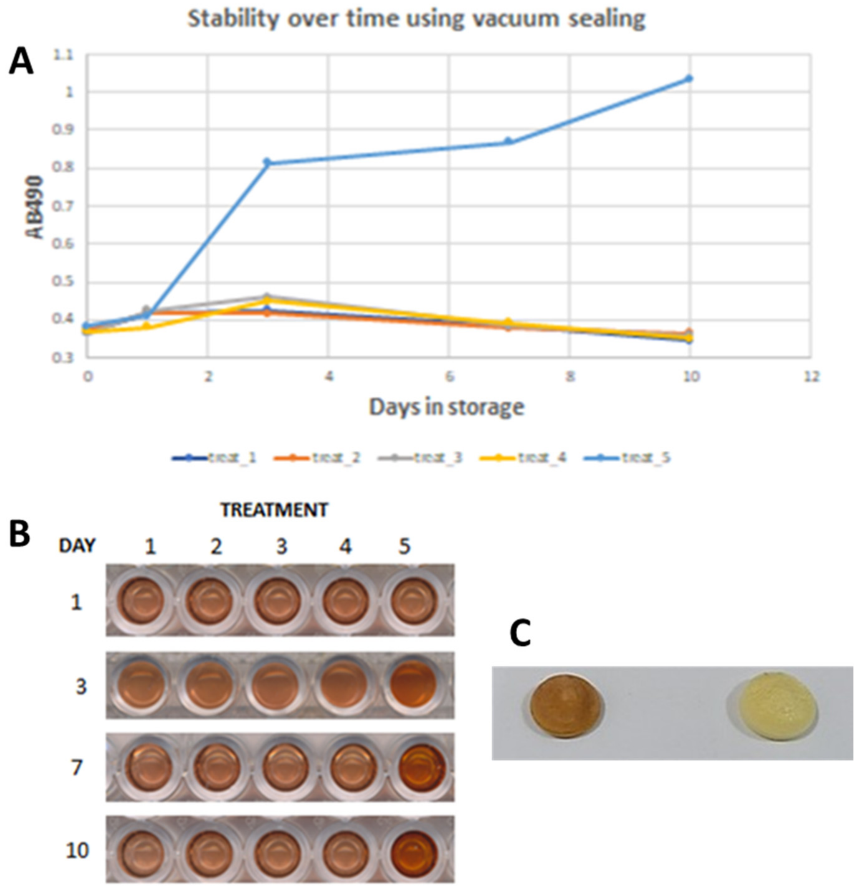

Figure S2. *Creatinine reagent stability. (A) Background absorbance (Ab490) of the lyophilized reagent versus days in storage; one inadequately protected condition (upper trace) developed a premature color, while the vacuum-sealed conditions remained low and flat. (B) Microplate time-course (days 1, 3, 7 and 10; five treatments) showing accelerated color development under heat and humidity. (C) Representative reconstituted reagent disks: left, spoiled (dark brown); right, intact (pale yellow).*

S 1.2.2. Hippuric acid reagent stability

The chemistry of hippuric acid demonstrated the greatest stability among the three tested. After 7 days at 45 °C and 70% relative humidity, color formation was consistently observed for all reagent components. When using undiluted standards, the color produced with stressed p-TsCl, whether non-vacuum-sealed (S) or vacuum-sealed (VS), was indistinguishable from that produced with fresh p-TsCl (G) in both water and urine (Figure S3A). A slight color difference between fresh and stressed p-TsCl was observed only with 10× diluted standards (Figure S3B); however, stressed

hippuric acid standards showed no difference from fresh ones. Reagents that were shipped to and returned from Nigeria performed effectively, with residual variability primarily due to pipetting rather than reagent degradation (Figure S3C). Given that the only detectable effect was a minor color shift related to p-TsCl at high dilution, it was recommended to generate a region-specific calibration curve the under local conditions in Nigeria to account for this variation.

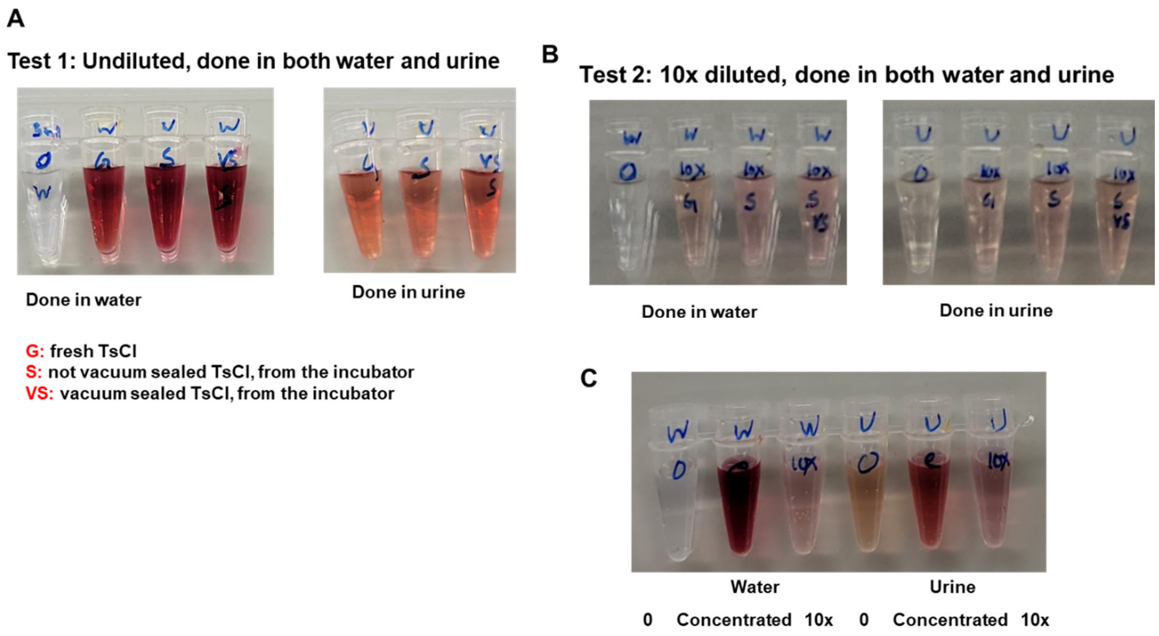

Figure S3. *Hippuric acid reagent stability after 7 days at 45 °C / 70% RH. (A) Undiluted standards in water (left rack) and urine (right rack) using fresh (G), non-vacuum-sealed stressed (S), and vacuum-sealed stressed (VS) p-TsCl; the color is indistinguishable across conditions. (B) The 10× diluted standards in water and urine, where a slight color difference between fresh and stressed p-TsCl is visible. (C) Independent repeats (two operators) using reagents shipped from Nigeria, across blank (0), concentrated, and 10× diluted samples in water and urine.*

S 1.2.3. Diacetylspermine reagent stability

The diacetylspermine assay demonstrated the highest environmental sensitivity among the three assays, attributable to its reliance on enzyme activity. When vacuum-sealed, both the dried enzyme/chromogen reagent and the diacetylspermine standard maintained stability for approximately 2–3 months at room temperature, 4 °C, and −20 °C, preserving the anticipated pink color upon reaction (Figure S4A). Vacuum sealing proved essential; under accelerated stress

conditions (45 °C / 70% RH), the vacuum-sealed batch, which was dispatched to Nigeria, withstood an 8-day exposure, whereas a freshly prepared, non-vacuum-sealed batch failed to develop color (Figure S4B). Following international shipping, the reaction remained operational; however, the diacetylspermine standard exhibited partial degradation. The reaction product from a standard returned from New York yielded an OD570 of 0.242 compared to 0.416 for an equivalent standard stored in Edmonton, indicating an approximate 40–50% reduction (Figure S4C). Also, tubes returned from the field showed enzyme pelleting, which took several minutes to dissolve again, and fully saturated (green) desiccant, both indicating that humidity entered during field transport. Despite this, sustained extreme stress surpassed the packaging's protective capacity; under continuous exposure to 45 °C / 70% RH, diacetylspermine activity was completely lost by 16 days (Table S1, case 6). The comprehensive stability dataset for the diacetylspermine assay is detailed in Table S1.

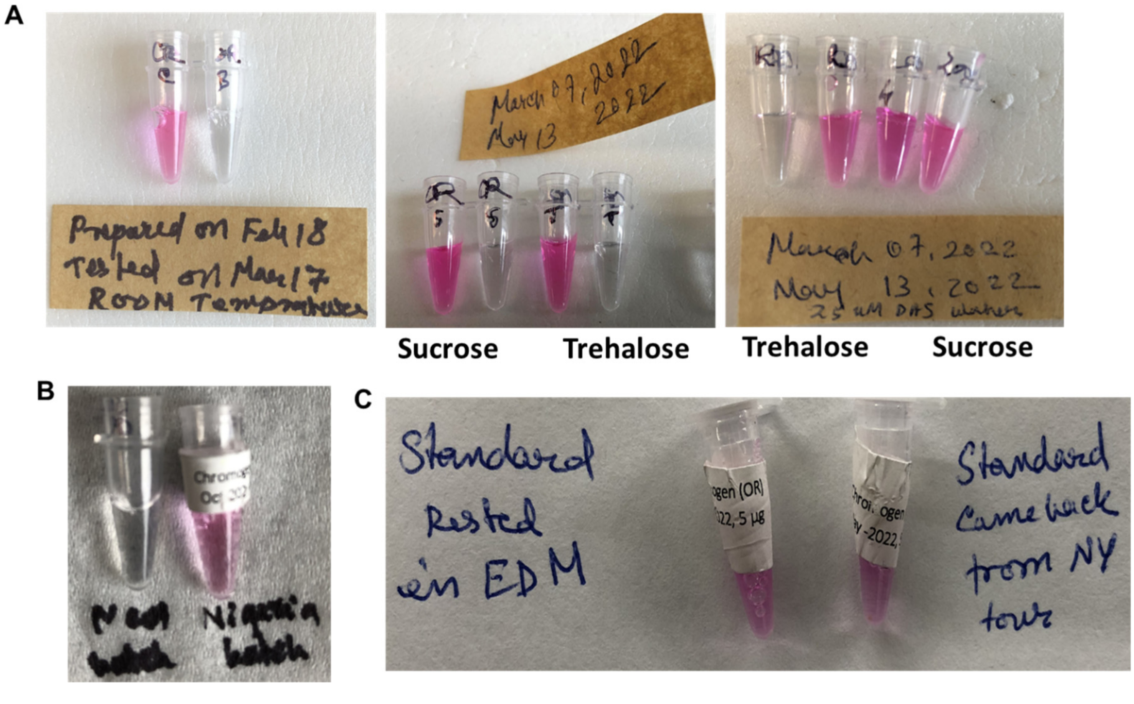

Figure S4. *Diacetylspermine (DAS) reagent stability. (A) Vacuum-sealed reagent stored at room temperature (~84 days), 4 °C and −20 °C (~68 days), with sucrose and trehalose stabilizers; the pink reaction color is retained. (B) Accelerated stress (45 °C / 70% RH, 8 days): The vacuum-sealed batch sent to Nigeria (right, pink) survived, whereas a non-vacuum-sealed batch (left) failed. (C) Effect of international shipping on the diacetylspermine standard: The standard held*

in Edmonton (left) versus the same standard returned from New York (right); the latter is fainter (OD570 0.242 vs 0.416).

Table S1. *Comprehensive summary of diacetylspermine assay stability across storage, shipping, and accelerated-stress conditions. All conditions used the optimized vacuum-sealed Mylar packaging with desiccant and oxygen absorber.*

Case	Location	Temperature	Relative humidity	Transport	Duration	Outcome
1	Edmonton (RT)	20–30 °C	~20–50%	No	84 d (~3 mo)	**Stable**
2	Nigeria	25–45 °C	~30–80%	Yes (shipped)	~4 mo	Active; ~50% of Edmonton activity retained
3	New York	Not recorded	Not recorded	Yes (shipped)	Not recorded	Reaction stable; standard degraded ~50%
4	Edmonton	4 °C (constant)	Not recorded	No	68 d (~2 mo)	**Stable**
5	Edmonton	−20 °C (constant)	Not recorded	No	68 d (~2 mo)	**Stable**
6	Edmonton	45 °C (controlled)	70% (controlled)	No (accel. stress)	16 d	**No activity**

"Not recorded" indicates a parameter that was not logged for that condition. Case 6 is the controlled-incubator accelerated-stress test (reagents were not transported). Case 2 retained roughly half the activity measured for the equivalently aged Edmonton reagent, and Case 3 showed an intact reaction with a partially degraded standard.

S 2: Workstation information

Peristaltic pumps: Peristaltic pumps were created using a combination of custom 3D-printed parts, stepper motors, tubing and parts from commercial peristaltic pump assemblies. Custom rollers/drums were designed and printed to provide consistency, including a peristaltic pump

housing with custom 3D-printed rollers/barrel that connected to the motor; each one was calibrated individually and had an error of ~+/- 50uL.

Dilution carousel: The dilution carousel used a nema stepper motor to rotate four 15mL Falcon tubes under one of two nozzles (one for water, one for urine). One Falcon tube was used for priming/cleaning the system (a waste tube) and the other three were for the three assays. The system was homed using a magnet and hall-effect sensor and using defined microsteps with the stepper motor; the carousel could achieve 0.1mm accuracy (well within the range to get the Falcon tube under the correct nozzle).

Elution tower: The elution tower was a vertical string pump with custom holders/connectors for the different resin syringes and syringe filters. The system used a stepper motor, screw, and three rods on linear bearings along with a physical limit switch for homing. Testing focused on constant flow rates and procedures to fully clear fluid from the resins. Experimentally, the elution tower could provide consistent flow rates of less than one drop every 3 seconds as required for the diacetylspermine assay. Tests were done to compare manual and automated elution by testing the percent recovery to DAS (measured by NMR) (Figure S5). While the % recovery was about 60% of that when done by an expert, the variability was less than that if done manually by less experienced individuals. The variability in elution by the device was found to be less than 10%, and the overall elution percentage was factored into creating the calibration curve used for the diacetylspermine assay for the system.

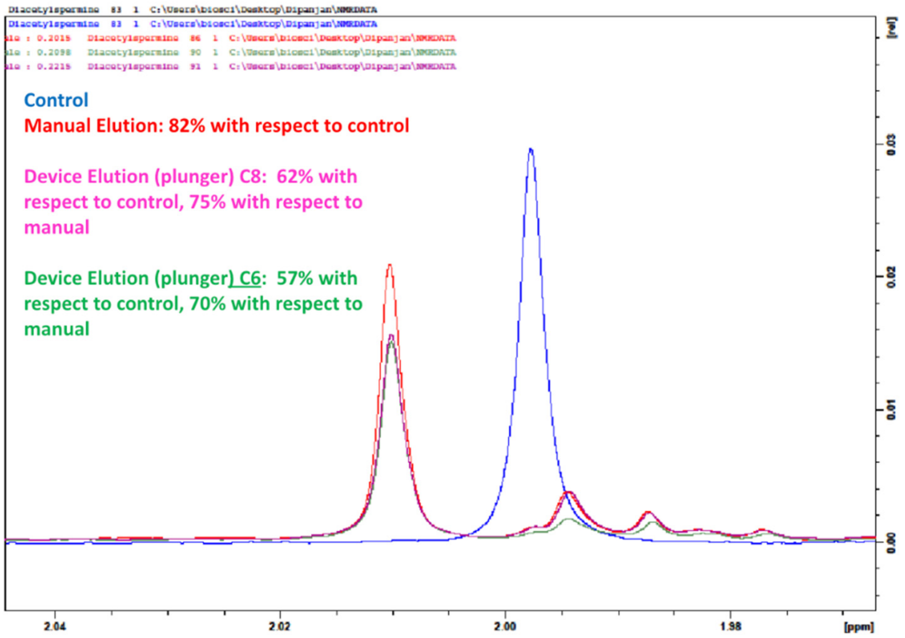

Figure S5: Automated elution system tested by NMR.
